# Bibliography

**Published:** 1901-07-15

**Authors:** 


### BIBLIOGRAPHY.

THE J. B. Lippincott Company announce a work by John S. Marshall, M.D., D.D.S., on the "PRINCIPLE AND PRACTICE OF OPERATIVE DENTISTRY." It will embrace a comprehensive discussion of both the theoretical and practical aspects of this subject. Dr. Marshall usually does things well, and while there seems no urgent demand for a work of this kind at present, we have no doubt it will be a welcome addition to our literature.

"INTERROGATIONS IN DENTAL METALLURGY," is a little book in a red cloth cover, published by the Calumet Publishing Company, of Pittsburg, Pa., and compiled by J. H. Beal, Sc. D., Professor of Metallurgy in the Pittsburg Dental College. The book asks nearly four hundred questions about the several metals usually employed in dental practice. The questions are well selected and we doubt not it might prove quite helpful in doing class work in this subject. Price 60 cents.

"ORAL SURGERY" is the title of a small hand-book on general medicine and surgery adapted to the needs of dental students, and prepared by Dr. S. L. McCurdy, A.M., M.D., Professor of Anatomy and Surgery in the Pittsburg Dental College, and published by the Calumet Publishing Company, of the same city. While the work contains less than four hundred pages, it covers very well all the field of interest to the dental practitioner, and as a text-book for dental students it has the advantage of being concise and yet sufficiently ample to serve as a basis for a course of lectures. The work is sufficiently well illustrated to aid the text in explaining the fundamental principles of the more complicated operations. The chapter on fractures of the maxilla is very well written and is well illustrated with cases from the author's clinic. It is a work in fact that dental practitioners might consult with very great benefit. Price $3.00.

---

THE fourth edition of "ESSIG'S DENTAL METALLURGY" has been issued from the press of Lea Brothers & Co. It has been brought down to date and re-edited so that it is probably the best work of its kind. There is not much change from former editions as these were carefully compiled. The subject of amalgam alloys has been carefully revised and includes the results of the investigations of Dr. Black. There is nothing to criticise and little to be desired. It is a standard work, and we have no doubt it will continue to command the endorsement of teachers and practitioners.

---

A NEW edition of "THE AMERICAN TEXT-BOOK OF OPERATIVE DENTISTRY," edited by Edward C. Kirk, D.D.S., and contributed by some seventeen authors has recently appeared. It contains eight hundred and fifty pages, one hundred and fifty more than the first edition. It is published by the well-know book house of Lea Brothers & Co., and is well done. Perhaps the most important addition is a chapter on the histological structure of enamel and dentin in their relations to cavity preparation. The article is well written and beautifully illustrated by numerous cuts, and is contributed by Dr. F. B. Noyes, of Chicago. A new chapter is also contributed by Dr. James Truman on sterilization of instruments and appliances in dental practice ; a very important subject and well treated. The chapter on porcelain inlays has been rewritten by Dr. Joseph Head, and is an up-to-date presentation of at least one aspect of this popular method of artistically supplying lost tooth structures. Almost every chapter has been revised or amended. The chapters devoted to orthodontia have been materially amended in both text and illustration, so much so in fact that it is very questionable whether it would not have been wiser to have left them out of the work altogether, especially since the work has become so voluminous as to give it more the character of a reference work than a text or hand-book for students. This is liable to be the result of all efforts to make a text-book from contributed articles. Dr. Kirk has succeeded well in his selection of contributors and in editorially classifying them ; but still there is a suspicion of lack of continuity in style and method which is not favorable to the development of that personal confidence and loyalty which one likes to feel toward a book whose teachings he desires to follow or whose authority he concedes.

